# Microscope image based fully automated stomata detection and pore measurement method for grapevines

**DOI:** 10.1186/s13007-017-0244-9

**Published:** 2017-11-08

**Authors:** Hiranya Jayakody, Scarlett Liu, Mark Whitty, Paul Petrie

**Affiliations:** 1 School of Mechanical and Manufacturing Engineering, UNSW, Sydney, Australia; 2 The Australian Wine Research Institute (AWRI), Adelaide, Australia; 3 South Australian Research and Development Institute (SARDI), Adelaide, Australia

**Keywords:** Stomatal morphology, Automatic stomata detection, Cascade object detection, Image processing, Skeletonization, Machine learning, Stomata, Grapevines

## Abstract

**Background:**

Stomatal behavior in grapevines has been identified as a good indicator of the water stress level and overall health of the plant. Microscope images are often used to analyze stomatal behavior in plants. However, most of the current approaches involve manual measurement of stomatal features. The main aim of this research is to develop a fully automated stomata detection and pore measurement method for grapevines, taking microscope images as the input. The proposed approach, which employs machine learning and image processing techniques, can outperform available manual and semi-automatic methods used to identify and estimate stomatal morphological features.

**Results:**

First, a cascade object detection learning algorithm is developed to correctly identify multiple stomata in a large microscopic image. Once the regions of interest which contain stomata are identified and extracted, a combination of image processing techniques are applied to estimate the pore dimensions of the stomata. The stomata detection approach was compared with an existing fully automated template matching technique and a semi-automatic maximum stable extremal regions approach, with the proposed method clearly surpassing the performance of the existing techniques with a precision of 91.68% and an F1-score of 0.85. Next, the morphological features of the detected stomata were measured. Contrary to existing approaches, the proposed image segmentation and skeletonization method allows us to estimate the pore dimensions even in cases where the stomatal pore boundary is only partially visible in the microscope image. A test conducted using 1267 images of stomata showed that the segmentation and skeletonization approach was able to correctly identify the stoma opening 86.27% of the time. Further comparisons made with manually traced stoma openings indicated that the proposed method is able to estimate stomata morphological features with accuracies of 89.03% for area, 94.06% for major axis length, 93.31% for minor axis length and 99.43% for eccentricity.

**Conclusions:**

The proposed fully automated solution for stomata detection and measurement is able to produce results far superior to existing automatic and semi-automatic methods. This method not only produces a low number of false positives in the stomata detection stage, it can also accurately estimate the pore dimensions of partially incomplete stomata images. In addition, it can process thousands of stomata in minutes, eliminating the need for researchers to manually measure stomata, thereby accelerating the process of analysing plant health.

## Background

Microscopic study of leaf epidermises aid researchers to gain a better understanding on the overall behavior and health of plants [1]. A microscope image of a leaf epidermis can provide a clear view of guard cells, epidermal cells, stomata and plant leaf veins. Among these elements, stomata, surrounded by guard cells, play a major role in protecting the plant against water loss and regulating the gas exchange with the external environment [2, 3]. As a result, the behavior of stomata provides key information on the water stress level, food production rate and the overall health of the plant [1, 4–6]. In an agricultural scenario, analysing stomatal behavior can lead to better resource management and yields [7, 8].

However, examining stomatal behavior from a microscope image is not a straightforward task. Different plants have different leaf structures, and biologists with expert knowledge are required to correctly identify and measure stomatal morphology. Currently, the most common approach to achieve this goal involves manual measurement of stomata pore dimensions using softwares such as ImageJ^®^ [9]. These type of tools require the user to manually mark the points of interest such as pore boundaries, stoma length and width so that the tool can produce the relevant measurement results. ImageJ^®^ also provides additional plugins in order to make tasks such as stomata identification easier, but users still need to manually tune parameters for each image to achieve reasonable results [10–12]. Even with the aid of such tools, the process of manually measuring stomata morphology is both time consuming and cumbersome. Due to the time constraints imposed by manual measurements, biologists are forced to select only a few stomata for measurement from each captured microscope image, and build statistical relationships and models using fewer data-points [13]. However, more robust statistical models can be built if all available data are measured. The solution therefore, would be to develop a fast, fully automated method which can accurately measure stomatal morphological features without any human intervention.

Several studies can be found on automatic detection and measurement of stomatal morphology. One of the first studies to investigate the possibility of automatically measuring stomata pore features was conducted by Omasa and Onoe [14]. In this research, a Hanning filter alongside a series of morphological operations is utilized in measuring the pore opening of sunflower stomata. However, this approach does not focus on correctly identifying stomata from a large microscope image in the presence of other background elements such as veins and dust particles. Instead, this method requires the input to be an image containing a single stoma. The work presented by Karabourniotis et al. [15] applies UV radiation to leaves, which as a result causes guard cells to emit a blue florescence. The plant leaves are then captured using a fluorescent microscope and the resulting images are filtered and segmented to extract stomata and guard cells. Even though this method produces reliable results, it requires a relatively featureless background as well as methods of applying UV radiation to the leaf. In addition, the work presented by Sanyal et al. uses image processing techniques on microscope images to classify different tomato types based on stomata structure [16]. A watershed technique is employed to extract a single stoma from a nearly featureless background. However, the proposed method would not perform well in the presence of multiple stomata and a feature-rich background.

More sophisticated approaches which aim to extract and measure stomata from feature-rich backgrounds can be found in the researches conducted by Laga et al. [13] and Liu et al. [17]. The work presented by Laga et al. [13] follows a template matching approach to identify and measure the stomata pore opening of wheat plants. Wheat has a very consistent leaf epidermal structure with wheat stomata roughly aligned in the same direction, which makes it suitable candidate for template matching. However, for irregular leaf structures this method requires more templates, and has the tendency to produce false positive results especially when there are vein structures which look similar to stomata. Furthermore, the stoma pore detection approach used in this research assumes that both the stoma and the guard cell boundaries are clearly captured by the microscopic image. However, in a practical scenario, the images captured are not perfect, and contain plenty of partially captured stomata. More recent research conducted by Liu et al. [17] focuses on detecting and measuring grapevine stomata by utilizing maximum stable external regions (MSER). Although less time consuming than using the ImageJ^®^ tool, this semi-automatic method still requires the users to interactively choose correct results from a given image and manually tune a set of parameters for each image. In addition, this approach always identifies stomata pore openings as symmetric ellipses, which is not the case in reality.

In this paper, we aim to develop a fully automated method to identify and measure stomata pore dimensions of grapevines, using microscope images. The images are prepared by applying a layer of resin and nail polish onto the leaf surface, and then carefully removing the nail polish layer which carries an imprint of the leaf epidermis. The final microscope image is generated by placing the nail polish impression on a microscope slide. The microscope images used for this research contain feature-rich backgrounds and the quality of the images captured vary depending on external conditions. Unlike previous work, where classical image processing techniques are used, the authors of this paper have opted to adopt a machine learning based cascade object detector to identify the stomata in a microscopic image. A similar cascade classifier has been previously applied to estimate the density of stomata in oak leaves [18]. However, compared to the work in [18] which uses Haar-like features for classification, the work presented in this paper utilizes HOG features to build the cascade object detector. Using a HOG descriptor, which is known to perform well in capturing the overall shape of an object, allowed the authors to build an accurate classifier using a less number of training samples (550 positive samples and 210 negative samples) compared to the work in [18] (10,000 positive samples and 3000 negative samples). It will be later shown that the training time required for a HOG based classifier is drastically lower compared to the Haar based COD proposed in [18] which took several days to train. A lower training time allows researchers to easily modify the proposed approach to train for different plant types with limited computing resources.

Once the stomata are automatically identified using the proposed COD algorithm, these regions of interest are cropped out and segmentation and skeletonization techniques are applied to the cropped image in order to measure the stoma pore boundary. Contrary to existing methods which require sharp, clean microscopic images for processing, the proposed approach, with the help of skeletonization, can estimate the stoma pore boundary under imperfect conditions where the stoma and guard cell boundaries are not fully visible, due to errors in applying resin, peeling off the nail polish layer etc. Here, skeletonization refers to the process of reducing a region to a skeletal remnant whilst preserving the connectivity features of the original image [19, 20]. The final result is a fully automated start-to-end stomata detection and measurement solution, where the input is a microscopic image of varying quality, and the output a set of stomatal morphologies.

The performance of this two stage method is then compared with the MSER method proposed by Liu et al. [17] and template matching method proposed by Laga et al. [13] using 50 microscopic images of cabernet sauvignon. Results show that the proposed approach is able to identify stomata more reliably, and produces accurate results in measuring the stomata pores.

The paper is organized as follows. In the “Methods” section, the image processing and machine learning techniques used to identify and measure stomatal properties are discussed in detail with examples. The experimental results of the study and comparisons with existing methods are presented in the “Results” section. The last section concludes the paper.

## Methods

The main aim of this work is to develop a fully automated solution for stomata measurement, where a microscopic image is used as the input to the system and the corresponding morphological features of the stomata in the image are treated as the final output. The proposed methodology consists of two stages. The first stage aims at correctly identifying the stomata in a given microscopic image. Once, the stomata are automatically identified and cropped out from the original image, the second stage analyses and measures the morphological features of each individual stoma. The steps involved in both of these stages are discussed in detail from the next section onwards.

### Cascade object detection algorithm to identify regions of interest

Cascade object detection (COD) algorithm is a multi-stage classification learner, where each stage is made up of a collection of weak learners. Each of these stages are trained using a technique called boosting. For the work presented in this paper, a COD which uses the Viola–Jones algorithm for face detection is re-trained for the purpose of identifying stomata [21, 22]. The COD approach inherently assumes that a large percentage of the image does not contain an object of interest. This in fact serves well for the question at hand, where the area covered by the stomata is small compared to the overall microscopic image area.

**Fig. 1:**
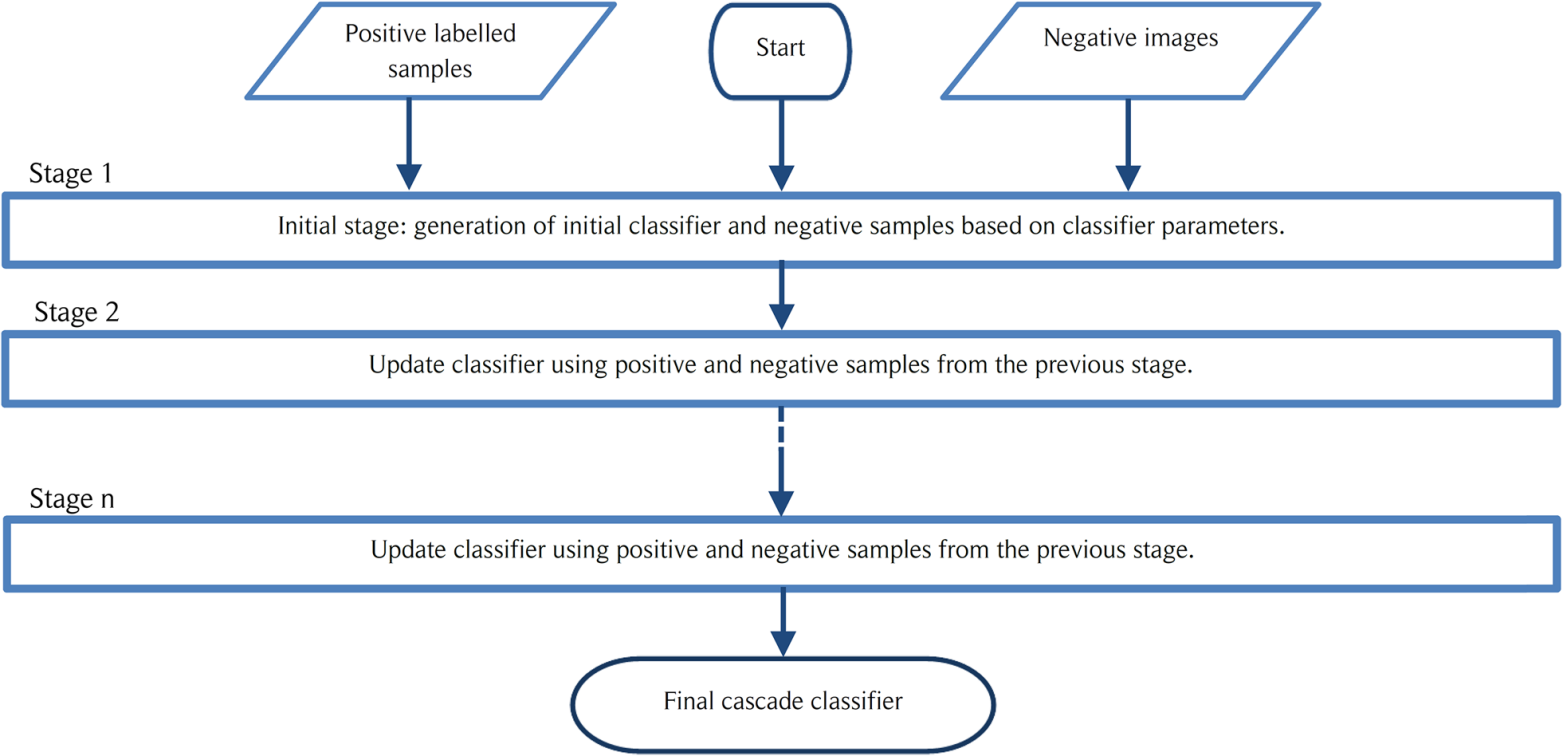
The operational procedure of an n-stage cascade classifier

**Fig. 2:**
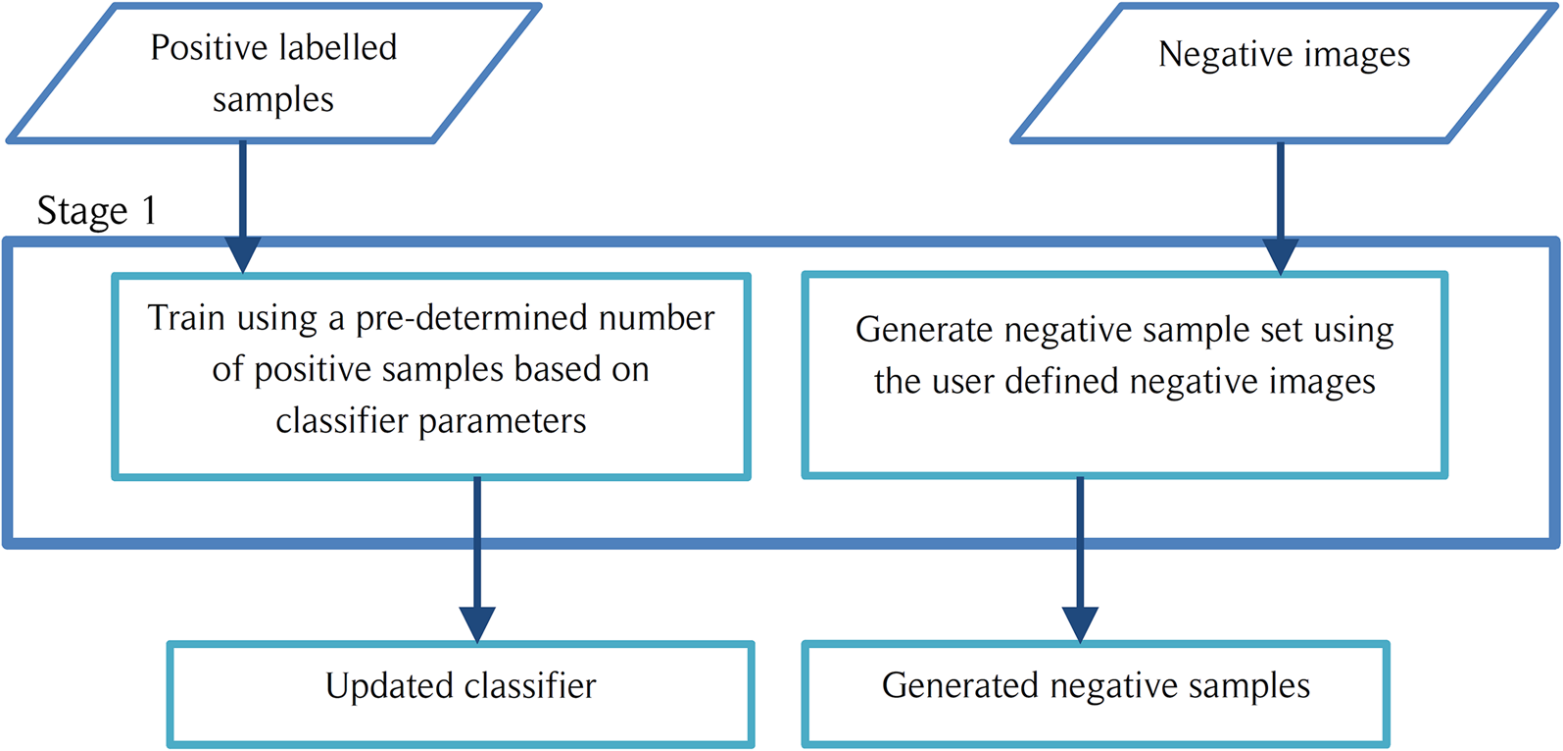
Function of the initial stage of a cascade object detector

**Fig. 3:**
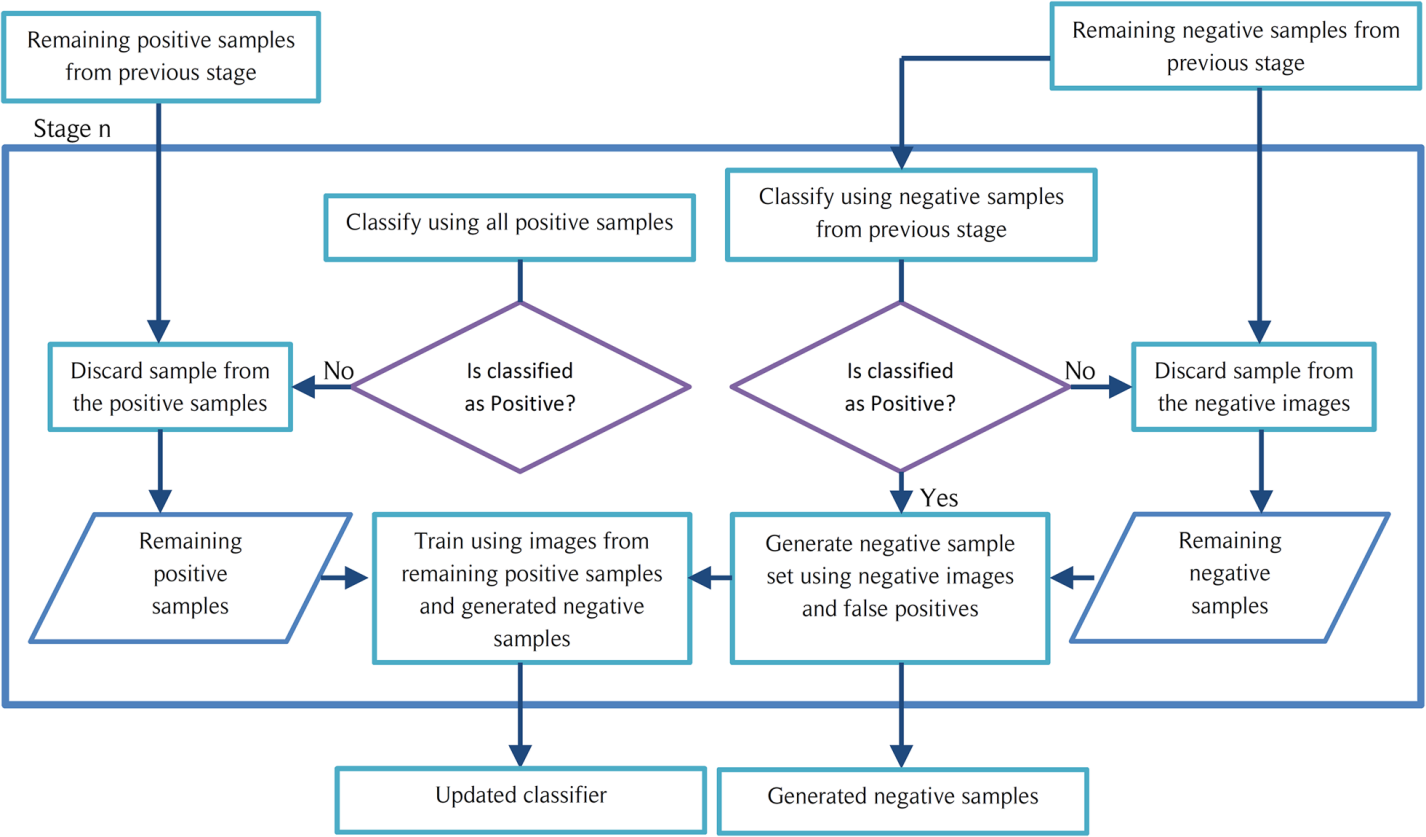
Function of a general stage of a cascade object detector

The COD approach is also known for reliably classifying objects of which the aspect ratio doesn’t change drastically. Furthermore, this method is better suited for situations where there are no out of plane rotations of the object. Thus, COD can be identified as a good candidate for the stomata detection since all stomata lie on a 2D plane and have minor aspect ratio changes. Also note that the COD method employed for this task uses Histogram of Oriented Gradients (HOG) as the main learning descriptor [23]. The implementation procedure for the COD algorithm consists of two major steps.

1. Train the cascade object detection classifier using a set of positive images (images containing stoma) and a set of negative images (images of veins, dust particles and other features). The overall simplified operational procedure for an *n* stage cascade classifier is presented in Fig. 1. A detailed representation of the operations carried out by the initial stage and a general stage of the classifier are shown in Figs. 2 and 3 respectively.
2. Slide a window over the microscope image and use the trained COD classifier to check for a stoma inside the window. If a stoma is detected inside the sliding window, define that area as a region of interest (ROI).

**Fig. 4:**
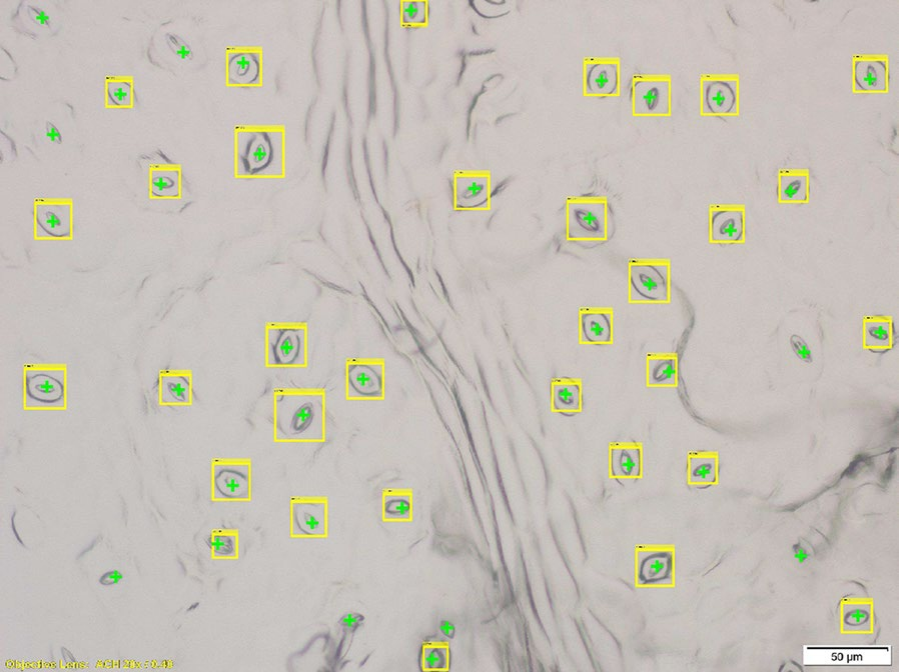
A sample result of the COD based stomata detection method. The green crosshairs represent actual stomata. The yellow bounding boxes show automatically detected regions of interest (ROIs)

Figure 4 shows the COD classifier at work. The bounding boxes which contain stoma are cropped and then sent to the second stage where binary segmentation methods alongside skeletonization techniques are applied to measure the pore morphology.

### Stomata pore measurement via binary image segmentation and skeletonization

**Fig. 5:**
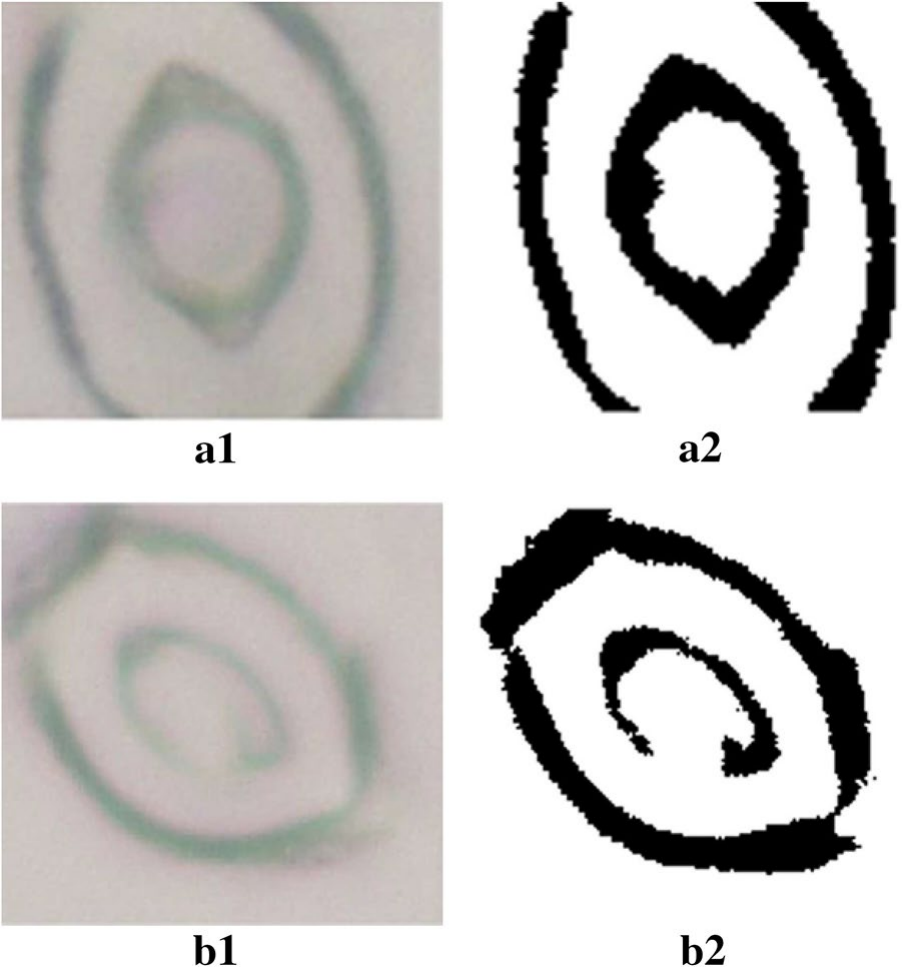
Examples of stomata captured with varying quality. **a.1** Stoma with a complete pore boundary. **a.2** Binary segmentation result for a complete pore boundary. **b.1** Stoma with an incomplete pore boundary. **b.2** Binary segmentation result for an incomplete pore boundary

Once the ROIs are identified and cropped, the next step is to detect and measure the stomatal pore in each ROI. Before proceeding with the pore measurements, it is important to observe the nature of the stoma captured. A closer look at the ROIs indicate that the stomata observed can be categorized into two types as,

1. Stomata with complete pore boundaries (see Fig. 5a.1).
2. Stomata with incomplete (discontinuous) pore boundaries (see Fig. 5b.1).

In order to develop reliable statistical models and relationships involving leaf epidermises, it is important to collect as much data as possible from a given microscope image. To the best of our knowledge, all previous research inherently discard stomata with low quality and require sharp, clean, complete boundaries in order to derive pore measurements. In this work, a skeletonization based approach is proposed to overcome this issue and estimate pore boundaries for low quality stomata with discontinuous pore boundaries.

The stomatal pore measurement stage has two sub-stages:

1. Binary image segmentation: estimates pore measurements for high quality, complete stomata.
2. Skeletonization and ellipse fitting: estimates pore measurements for low quality incomplete stomata.

First, all cropped stomata images are fed through the binary image segmentation method. The binary image segmentation method can accurately estimate the stomatal pore areas for high quality images. However, this method fails when processing low quality images with discontinuous boundaries. Therefore, whenever this method fails in identifying the stomatal pore area, the corresponding low quality image is then fed into the skeletonization and ellipse fitting method. Adopting such a method ensures that pore boundaries are identified for the majority of the stomata detected under varying image quality.

#### Binary image segmentation

The following set of steps are employed to estimate the stoma morphology for complete pore boundaries.

1. The image is sharpened, converted to grayscale and then converted to a binary image.
2. Independent regions (disconnected from each other) are identified on the binary image.
3. The region representing the stomatal pore opening is identified based on two assumptions: (a) the stoma is closer to the center of the ROI, (b) the pore area is smaller than a predefined upper limit. The upper limit of the pore area represents the approximate maximum area that can be covered by a stomatal pore. This parameter depends on the resolution and the zoom level of the microscopic image. The upper limit can be defined by briefly observing the original images and gaining an understanding on how large a typical stoma is (pixelwise).
4. The pore opening is marked and the morphological features such as area, major axis length, minor axis length and eccentricity are measured.

**Fig. 6:**
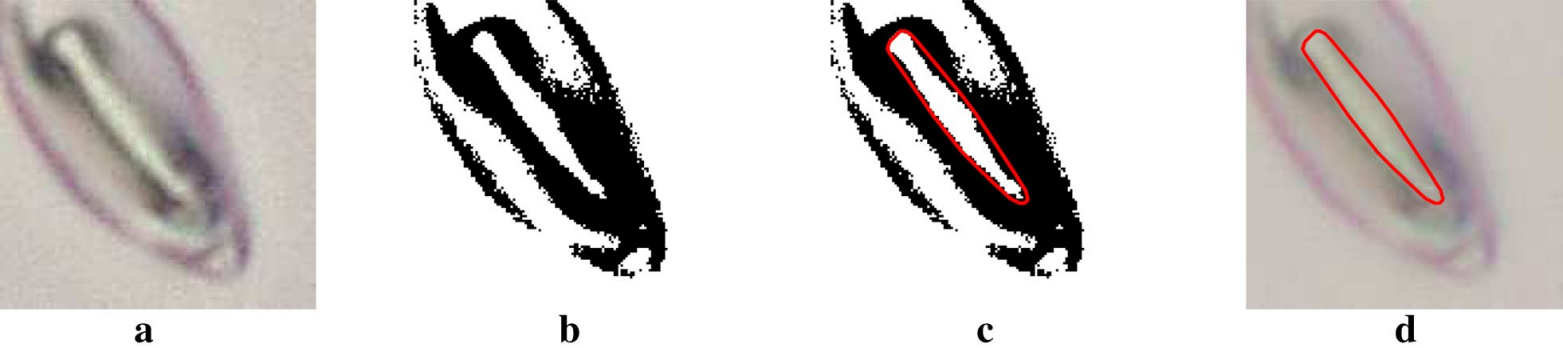
The binary image segmentation process. **a** Original image. **b** Binary image. **c** Identify pore region. **d** Pore boundary overlaid on the original image

A visual representation of this method is shown in Fig. 6. This simple approach produces reliable results when the stoma is of good quality. However, if the stoma pore boundary is discontinuous, the binary image of the stoma would not contain a independent region which agrees with the two assumptions made in step 3 (see Fig. 5b.2 for such a condition). Therefore, such images are discarded and handed over to the skeletonization and ellipse fitting method. A detailed description of the skeletonization approach is presented in the next section.

#### Skeletonization and ellipse fitting

Image skeletonization refers to the process of reducing a selected region to a skeletal remnant which represents the medial axis of that region [19]. The following set of steps are applied to the images discarded by image segmentation sub-stage, with the aim of estimating stoma morphological features in the presence of discontinuous pore boundaries.

1. The image is sharpened, converted to grayscale and then converted to a binary image.
2. Independent regions (disconnected from each other) are identified on the binary image.
3. The binary image is inverted.
4. The independent regions on the image are skeletonized (also known as deriving medial axes). Each skeletal remnant would be a vector containing pixel coordinates.
5. The skeletal remnant associated with the pore boundary is then identified based on two assumptions: (a) the skeletal remnant associated with the stoma is closer to the center of the ROI. (b) The length of the skeletal remnant lies between a pre-defined upper and lower limit.
6. Once the correct skeletal remnant is identified, generate an ellipse which fits the points of the skeletal remnant.
7. This ellipse is then used as a mask on the binary image derived in step 2. The independent region inside this mask is identified as the stoma pore.

**Fig. 7:**
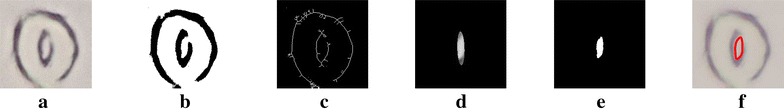
Skeletonization and ellipse fitting process. **a** Original image. **b** Binary image. **c** Derivation of independent line segments via skeletonization. **d** Fit ellipse to the skeletal remnant representing the pore opening. **e** Binarize the region inside the ellipse and identify regions. **f** Pore boundary overlaid on the original image

A visual representation of this step-by-step approach is shown in Fig. 7. Skeletonization and ellipse fitting, together with binary image segmentation ensures that morphological features are measured for a large percentage of the initially detected ROIs. Compared to the traditional approach of manually measuring stomata which drastically limits the number of stomata which can be measured, this novel approach provides a comprehensive solution which provides pore measurements for a large number of stomata in quick time.

## Results

The performance of the two stage stomata measurement method was compared with Liu’s MSER approach and Laga’s template matching approach. Programs for all three methods were developed using Matlab^®^ R2017a.

### Training procedure

**Fig. 8:**
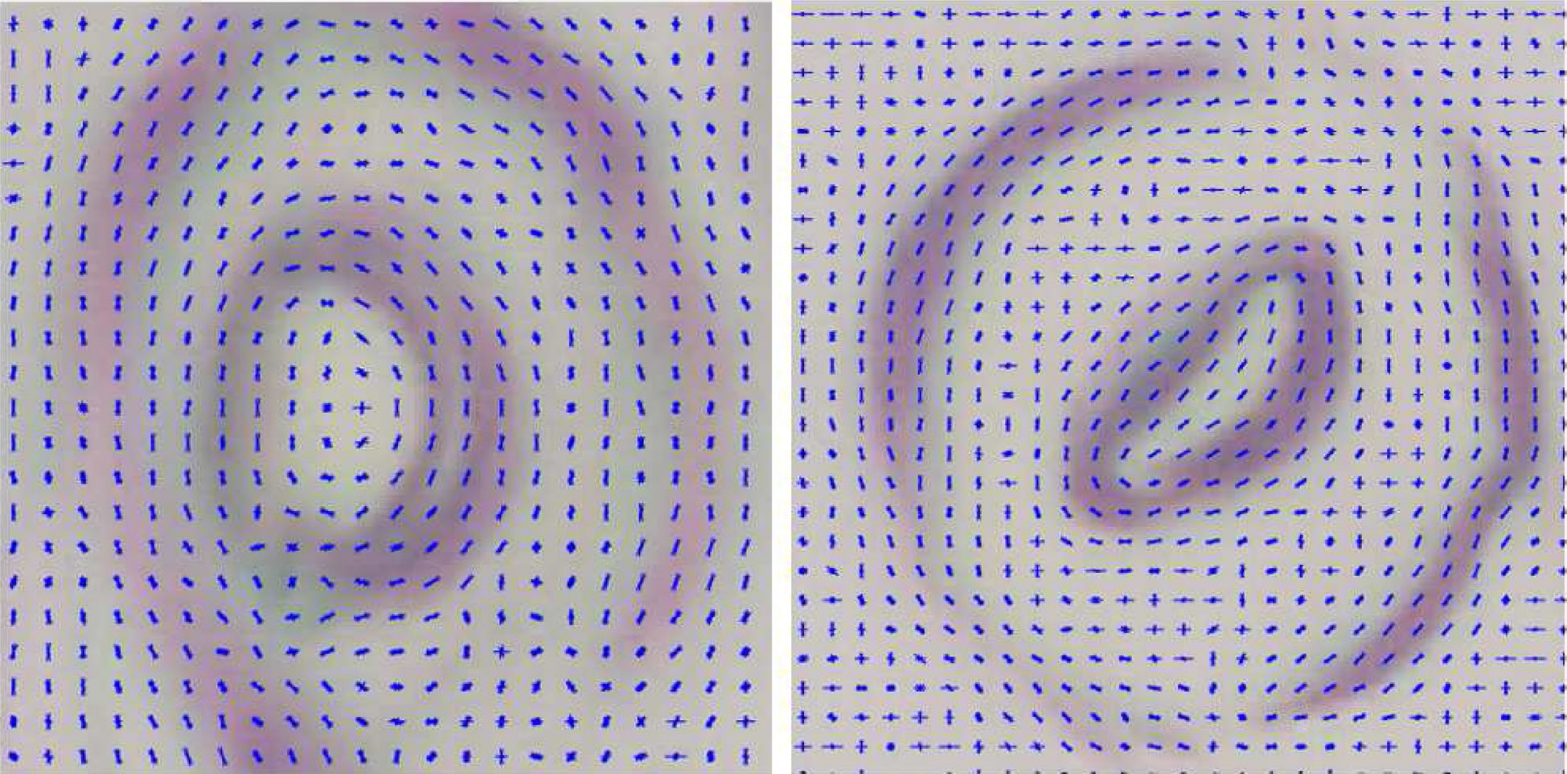
HOG feature visualization for positive samples

The training step of COD was conducted using 550 positive samples where each image contained a single stoma, and 210 negative samples which contained other leaf epidermis features such as veins and dust particles. The classifier consists of 8 stages, and utilizes HOG features as the main descriptor. The visual representation of the HOG features on positive samples are shown in Fig. 8. The training process took approximately 7 min, inside the Matlab^®^ environment on a 2.2 GHz Intel^®^ Core i7-4702MQ CPU with 16 GB RAM. Note that COD training with HOG features takes drastically less processing time compared to the classifier used in [18] which took several days to train.

### Data collection

The trained classifier was then tested on a separate 50 microscope images collected from cabernet sauvignon leaves containing 2012 stomata. The images were prepared using the conventional approach, where a layer of resin and nail polish are applied to the leaf epidermis, and an imprint of the leaf surface is captured by removing the nail polish layer and placing it on a microscope slide. The microscope images were captured using an Olympus^®^ DP73 camera attached to an Olympus^®^ BX53 microscope. The image resolution was set at 4800 × 3600 pixels, with a magnification of 8.6 pixels/μm.

### Stomata detection

The stomata detection capability of the proposed COD approach was put to test first. In order to measure the performance improvements of the proposed method, two other existing methods, namely, Laga’s template matching approach and Liu’s maximum stable extremal region approach, were applied to the same 50 images. Since Liu’s MSER approach is not a fully-automated method, we tuned the MSER parameters such that it provided best possible results for the given image set, and then automated the process in order to make the three methods more comparable. The template matching method was implemented using 20 stoma templates. Detailed implementation instructions for both template matching and MSER methods can be found in [13] and [17].

**Table 1:** Numerical results obtained for template matching, MSER and COD methods, using 50 microscopic images containing 2012 stomata

	Actual number of stomata	ROIs detected	True positive	False positive	False negative
Template matching	2012	2331	1324	1007	688
MSER	2012	1398	746	652	1266
COD (proposed)	2012	*1742*	*1597*	*145*	*415*

The numbers for the proposed method were italisized to emphasize the improvement of the proposed approach

**Table 2:** Statistical results obtained for template matching, MSER and COD methods, using 50 microscopic images containing 2012 stomata

	Precision (%)	Recall (%)	Accuracy (%)	F1-score
Template matching	56.64	65.50	43.95	0.60
MSER	53.36	37.08	28.00	0.44
COD (proposed)	*91.68*	*79.37*	*74.04*	*0.85*

The numbers for the proposed method were italisized to emphasize the improvement of the proposed approach

The corresponding results obtained after applying these three methods to 50 microscopic images are presented in Tables 1 and 2. The proposed method not only generated the highest number of true positives, it also resulted in the least number of false positives. Thus, the results clearly reflect the superiority of the the cascade classifier compared to the other two existing autonomous approaches. Further statistical analysis of the results showed that the proposed COD approach had the highest precision, recall and accuracy rates among the three methods (see Table 2). It is also the only method to surpass an F1-score of 0.80. The low number of false positive results generated by COD can be identified as the main reason contributing to this superior F1-score.

### Stomata measurements

The next step was to test the performance of the second stage of the proposed approach. In this stage, the main aim of the algorithm was to estimate the morphological features of the stomata pores. For this experiment, the 1742 ROIs detected through the COD method were used as the input. The corresponding results are presented in Table 3. Out of 1742 identified ROIs, the binary image segmentation method combined with skeletonization was able to generate results for 1267 stomata while discarding 475 ROIs. Further analysis showed that the 475 ROIs discarded by the pore estimation method included false positives generated by the COD as well as stomata of which the pore boundary could not be identified with any confidence, due to the image being out of focus or stoma being partially captured. Next, the generated 1267 estimations were visually inspected. These inspections showed that this approach was able to correctly identify the pore boundaries 86.27% of the time. The inaccurate results (174 out of 1267 ROIs) often identified the guard cell boundary as the stoma opening. However, this small number of inaccuracies does not pose a threat to the final result, as the user can easily visually inspect and remove such results from the dataset. It is important to note that the time spent on discarding inaccurate results via visual inspection is negligible compared to the time consumed in manually marking over a 1000 stoma pore openings.

**Table 3:** Results obtained for stomata pore estimations for 1742 ROIs

	Number of ROIs as input	Discarded ROIs	Accurate pore identifications	Inaccurate pore identifications	Identification accuracy
Binary image segmentation with skeletonization and ellipse fitting	*1742*	*475*	*1093*	*174*	86.27%

The numbers for the proposed method were italisized to emphasize the improvement of the proposed approach

**Fig. 9:**
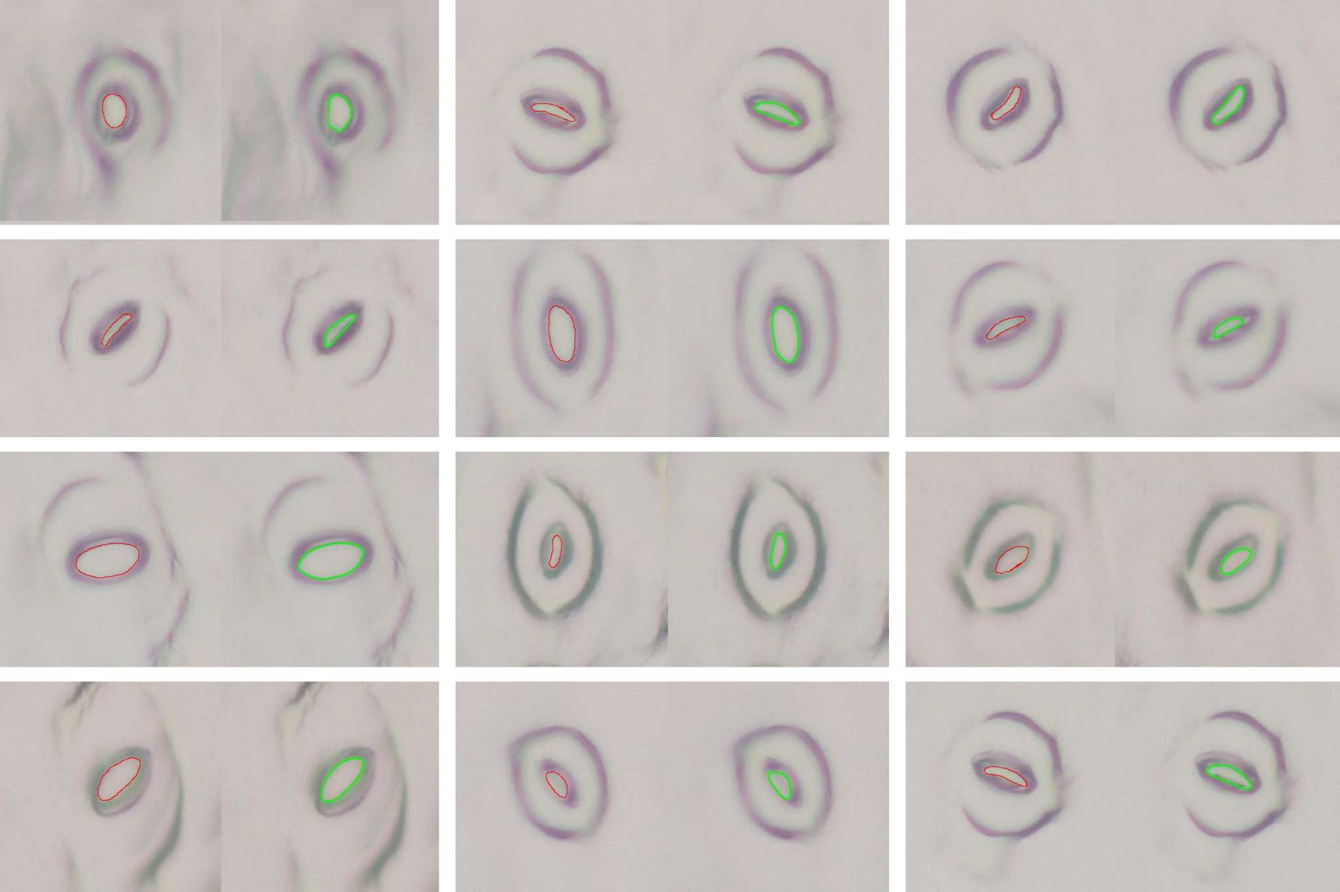
A sample segment of stomata pore measurement results. A red trace represents a manually marked (ground-truth) stoma pore. A green trace represents automatically measured pore for the same stoma

Let us now consider the correctly marked stomata. It is important to measure how the automatically generated stomatal pore measurements compare with manually marked stomatal pores traced using tools similar to ImageJ^®^. In order to make this comparison, the stoma boundary was manually marked under expert supervision for 70 randomly generated ROIs. These manually marked boundaries were considered as the ground truths. Then the manually measured parameters were compared with the measurements generated by the proposed automated method. The following equations were used to estimate the major axis length, *a*, and minor axis length, *b*,

1 $$\begin{aligned} a = \sqrt{\frac{A}{\pi \sqrt{1-E^2}}}, \end{aligned}$$

2 $$\begin{aligned} b = \sqrt{\frac{A \sqrt{1-E^2}}{\pi }}, \end{aligned}$$

where, *A* is the area of the stoma pore and *E* is the eccentricity of the detected pore. The corresponding results of the experiment are presented in Table 4. Here, the term accuracy is defined as,

3 $$\begin{aligned} {\mathrm {Accuracy}}\,(\%) = |(Y - \hat{Y})/Y|\times 100, \end{aligned}$$

where, *Y* is the actual value, and $\hat{Y}$ is the estimated value. According to the results, the pore area traced by the automated method is always slightly larger than the manually marked area but holds an accuracy reading of 89.03%. However, the eccentricity values are highly accurate as the errors in major and minor axis length measurements are quite uniform (i.e: similar estimation errors in *a* and *b* would not highly affect the term *b*/*a*). The average accuracies for both major axis length and minor axis length surpass 90%, with accuracy readings of 94.06 and 93.31% respectively. A side-by-side visual comparison between the ground truth and the estimation for 12 test images is presented in Fig. 9.

**Table 4:** Comparison of automatic stomatal pore measurements with manual measurements derived using ImageJ^®^

Number of stomata compared	Avg. area accuracy	Avg. eccentricity accuracy	Avg. major axis length accuracy	Avg. minor axis length accuracy
70	89.03%	99.43%	94.06%	93.31%

Observing the results, it can be concluded that the fully automated method is able to provide accurate morphological measurements for 1093 stomata out of 2012 available stomata in a small amount of time. Please note that the two stages together have discarded 890 stomata due to various reasons such as stoma being too blurry, not properly captured etc. The time consumed by an Intel i7 computer with 16 GB RAM to process the 50 images of high resolution (4800 × 3600 pixels) was measured to be 10 min (roughly 12 s to process 40 stomata). These results suggest that the proposed approach can save a huge amount of time in processing large sets of microscopic data, when compared to manual approaches.

## Discussion

As per the results, the proposed two stage fully automated method is able to out-perform existing stomata detection method as well as accurately measure stoma pore dimensions. The reasons which result in such an improvement are discussed next.

**Fig. 10:**
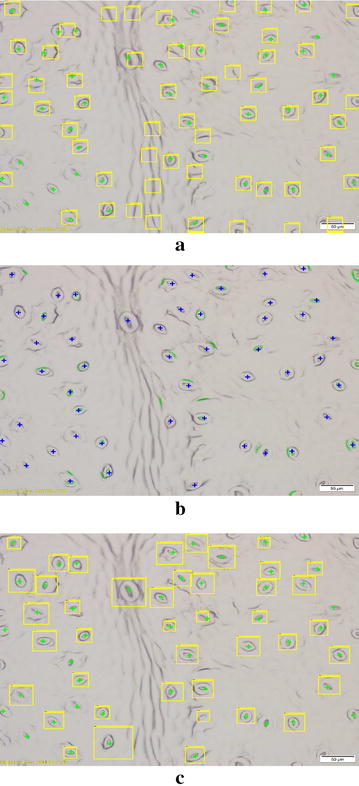
Stomata identification results for three different methods. **a** Result for Laga’s template matching method. **b** Result for Liu’s MSER method. **c** Result for the proposed COD method

Figure 10 shows the results generated by the three methods for a sample microscopic image. The template matching approach works well in highlighting areas containing stomata as shown in Fig. 10a. Note that this is the first time the template matching approach was applied to a leaf structure with stomata oriented in all directions. In this scenario, the template matching method is prone to highlighting other epidermal elements such as veins and dust particles which align well with some stomata and have similar thicknesses. This causes the template matching method to generate a high number of false positives. On the other hand, the MSER approach proposed by Liu et al. searches for stable elliptical regions in the image. Thus, their approach is not robust enough to differentiate between stoma pore openings, outer guard cell walls and veins containing elliptical patterns. This results in a high number of false positives as well. In addition, this method tends to discard stomata pores of which the interior is not stable enough for detection. These issues are clearly illustrated in Fig. 10b.

The proposed cascade object detection approach identifies stomata by learning their overall appearance. Thus, it is able to identify stomata in a more robust manner, whilst keeping the number of false positives to a minimum. However, this method too would ignore stomata which look considerably different from the training data set (e.g: blurred stomata, partially captured stomata). Furthermore, as a learning algorithm, the performance of the proposed cascade classifier is subject to change depending on the hyper-parameters (number of stages, number of false positives allowed per stage etc.) used during learning as well as the nature of the training dataset used. Special attention should be paid to the size and the features captured by the training datasets in order to produce the best possible results. This cascade classifier approach can successfully perform with a wide range of leaf types. However, the classifier would require re-training with suitable training data for leaf types with considerably different stomata or background structure.

Let us now consider the stomata pore measurement process. The proposed pore measurement methodology, which involves binary image segmentation combined with skeletonization and ellipse fitting, does not require stoma boundaries to be sharp and continuous like Laga’s template matching approach. It is fully capable of estimating stoma pore dimensions even in cases where the pore boundary is only partially visible in the image. However, in order to estimate the pore dimensions for a partially complete boundary, the boundary should be at least 60–70% complete. In other words, the implemented ellipse detection algorithm struggles to derive a confident estimate for boundaries which are more than 50% incomplete. This is one main reason for the stomata pore measurement stage to discard 475 ROIs from the 1742 detected ROIs (see Table 3).

## Conclusions

This paper presented a fully automated start-to-end solution for estimating stomatal morphological features of grape leaves. This two stage approach, which comprises of a cascade object detector to identify stomata in an image, and a combination of segmentation, skeletonization and ellipse fitting techniques to measure the stomata pore opening, was able to perform better than recently developed automated stomata detection methods. The COD approach identified stomata with a precision of 91.68% and an F1-score of 0.85. Out of the identified stomata, this approach managed to correctly trace the pore boundary of the stoma 86.27% of the time. Comparisons with ground truths show that the proposed approach measures the pore area with an accuracy of 89.03% the eccentricity with an accuracy of 99.43%. Compared to existing pore measurement methods, the proposed approach can estimate pore dimensions for stoma with incomplete pore boundaries. All the tests were conducted using grape leaves of type cabernet sauvignon. The authors intend to extend this research to test on different varieties of grapes and other plant types.

## Abbreviations

COD: Cascade object detection
HOG: Histogram of oriented gradients
MSER: Maximally stable extremal regions
UV: Ultra violet
